# Taking the Hinge off: An Approach to Effector-Less Monoclonal Antibodies

**DOI:** 10.3390/antib9040050

**Published:** 2020-09-23

**Authors:** Jamie Valeich, Dan Boyd, Manu Kanwar, Daniel Stenzel, Deblina De Ghosh, Arpa Ebrahimi, James Woo, Jenny Wang, Alexandre Ambrogelly

**Affiliations:** Pharmaceutical & Biologics Development, Gilead Sciences, 4010 Ocean Ranch Blvd, Oceanside, CA 92056, USA; Jamie.Valeich@bms.com (J.V.); Dan.Boyd@gilead.com (D.B.); Manu.Kanwar@gilead.com (M.K.); Daniel.Stenzel@gilead.com (D.S.); Deblina.DeGhosh@gilead.com (D.D.G.); arpa.ebrahimi@gilead.com (A.E.); jwoo02@gilead.com (J.W.); Jenny.Wang4@gilead.com (J.W.)

**Keywords:** monoclonal antibody, mAb, hinge, effector function, Antibody-dependent cellular cytotoxicity (ADCC), IgG4, IgG1, Fcγ receptors, FcγRI, FcγRIII, Fc neonatal receptor, FcRn, Fab arm exchange

## Abstract

A variety of Fc domain engineering approaches for abrogating the effector functions of mAbs exists. To address some of the limitations of the current Fc domain silencing approaches, we are exploring a less commonly considered option which relies on the deletion of the hinge. Removal of the hinge domain in humanized IgG1 and IgG4 mAbs obliterates their ability to bind to activating human Fc gamma receptors I and IIIA, while leaving their ability to engage their target antigen intact. Deletion of the hinge also reduces binding to the Fc neonatal receptor, although Fc engineering allows partial recovery of affinity. Engineering of the CH3 domain, stabilizes hinge deleted IgG4s and prevents Fab arm exchange. The faster clearing properties together with the pacified Fc make modality of the hinge deleted mAb an appealing solution for therapeutic and diagnostic applications.

## 1. Introduction

Gamma Immunoglobulins are key components of innate and acquired immunity. Their central role in controlling pathogen infections relies on two specialized functional modules: the antigen binding fragment (Fab) and the crystallizable fragment (Fc) [1]. The Fab is composed of the variable (Fv) and CH1 domains and is responsible for the recognition and binding to the target antigen via a set of six complementarity determining regions (CDRs) [1]. The Fc is composed of the CH2 and CH3 domains and mediates the interaction with the complement C1 complex and activating or inhibitory Fcγ receptors [1]. These Fc-mediated interactions enable the recruitment of effector cells and contribute to the potentiation and regulation of the immune response [2]. The Fc domain of immunoglobulins also binds to the neonatal Fc receptor (FcRn), an important component of the immunoglobulin endosome recycling process leading to prolonged half-lives [1]. Located between the Fab and the Fc domains, the hinge of human IgG1, 2 and 4 antibodies is a short linker peptide of 12 to 15 amino acids containing two to four inter heavy chain (HC) disulfide bridges [1]. The hinge domain of IgG3s is elongated and made of a repeat of four peptides homologous to that of the IgG1 hinge [1].

Small angle X-ray scattering, cryo-electron tomography and X-ray crystallography studies show that the hinge region of the four IgG subclasses is structurally disorganized and inherently flexible [3,4,5,6,7]. In a physiological context, hinge flexibility enables the antigen binding fragments to move freely relative to each other and to the Fc [6,7]. This critical feature facilitates the concomitant interaction of the antibody with up to two target antigens and the complement/Fc receptors [6,7]. The same structural studies also show that the extent of flexibility of the hinge varies between members of the four different IgG isotypes; the most flexible being the elongated hinge of the IgG3, followed by those of the IgG4, IgG1 and lastly IgG2 [1]. Differences in hinge flexibility are caused by variations in sequence as well as differences in inter heavy chains and heavy–light chain disulfide linkages.

The four human gamma chains subclasses show over 90% amino acid sequence homology, with most of the sequence variations concentrated in the hinge and amino terminus of the CH2 domain [1]. These sequence differences have important functional implications. The lower proline content of the short IgG4 hinge enables the facile isomerization of the interheavy chain disulfide bridges and is an important molecular determinant of bispecific antibody formation via the Fab arm exchange (FAE) process [1,8]. In contrast, the higher proline and cysteine content of the IgG2 hinge results in a complex network of hinge disulfide isomers directly responsible for the higher rigidity of this IgG subclass [1]. The resulting disulfide isomers were shown in some instances to have different affinities for their cognate antigen and different receptor agonist properties [9,10]. Lastly, the flexibility of the IgG1 hinge determined by sequence and disulfide linkages may be a key determinant of the allosteric communication between Fab and Fc [11].

The remarkably high rate of sequence divergence of the hinge and CH2 amino terminus also relates to the different ability of the four IgG isotypes to engage complement and the inhibitory or activating Fc gamma receptors [1]. Structural and biochemical studies have highlighted the presence of residues in the lower hinge of IgG1, IgG3 and to some extent in IgG2 antibodies, which are key to complement activating C1q protein binding, and the absence of these residues from the IgG4 framework [1]. Likewise, a large body of data has shed light on the molecular basis for the asymmetric binding of high affinity FcγRI across the lower hinge and proximal CH2 domain residues of IgG1, IgG3 and IgG4 antibodies [2,12,13]. These studies also showed the overall binding footprint of FcγRI to be shared in great extent with those of the low affinity FcγRII and III [2,12,13]. The rationale for the specificity of FcγRIIIa for IgG1 was elucidated and the unique ability of this antibody subtype to mediate antibody dependent cell cytotoxicity (ADCC) or cellular phagocytosis (ADCP) was explained [2,12,13].

Taking advantage of the thorough understanding of the interaction between FcγRs and the constant domain of IgG molecules, protein engineers have devised therapeutic molecules with enhanced efficacy and/or better safety profiles [14]. Enhancing the efficacy of mAbs relying on ADCC as part of their mode of action implies tightening the interaction with FcγRIIIa. This is achieved via point mutations in the lower hinge or glycoengineering of the conserved N-glycans in the CH2 domain [14,15]. In contrast, improving the safety profile by dampening the ADCC effector functions by lowering the affinity to FcγRIIIa can be achieved by selecting an IgG4 or IgG2 scaffold or by engineering an IgG1. The engineering solutions include insertion of point mutations in the CH2 domain or complete removal of the conserved Fc N-Glycan by modifying the consensus glycosylation site [14]. While these strategies may reduce ADCC, they do not necessarily abolish binding to complement or other Fc receptors to the same magnitude, and they may contribute to potential immunogenicity or thermodynamic instability of the Fc domain.

**Figure 1:**
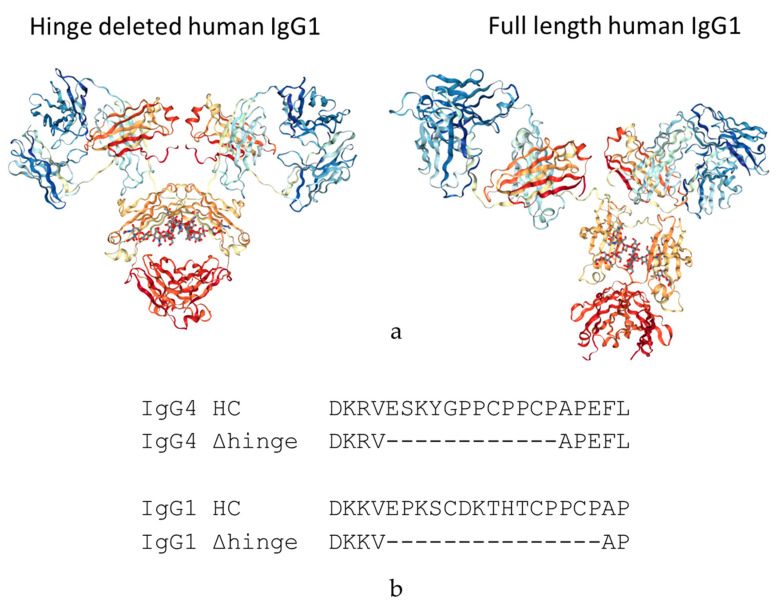
(**a**) Three-dimensional representation of the crystal structures of a human IgG1 (PDB 1HZH) and the hinge deleted Mcg Antibody (PDB 1MCO). (**b**) Sequence alignment of the IgG1 and IgG4 hinge (IgG1 HC, IgG4 HC) and resulting sequence after deletion (IgG1 Δhinge and IgG4 Δhinge).

We are proposing a different approach to pacify the Fc of therapeutic antibodies. This approach consists of the complete deletion of the hinge peptide from the heavy chain polypeptide and fusing the carboxy terminus of the CH1 domain to the amino terminus of the CH2 domain. Hinge deleted IgG1s have previously been described in Myeloma patients as the result of a genetic mutation. For these molecules, termed Dob and Mcg, the deletion of the hinge resulted in conformationally restricted antibodies with reduced or no effector function (Figure 1) [16,17]. More recently, a hinge-deleted IgG4 was proposed as a acetylcholine receptor blocker therapy in a model of the neuromuscular autoimmune disease myasthenia gravis in rhesus monkeys [18]. The present paper describes the generalization of the approach to different molecules as well as their biochemical and functional characterization.

## 2. Materials and Methods

### 2.1. mAb Production and Purification

The recombinant humanized hinge deleted and full length IgG1 and IgG4 mAbs used in this study were produced transiently in Chinese Hamster Ovary cell and a standard fed batch fermentation process. Cell culture harvests were clarified by centrifugation and filtration followed by capture on a Protein A column. Elution from the protein A column was performed using a sodium acetate buffer as per standard industry practices. The mAbs were further purified as needed by a subsequent gel filtration chromatography polishing step to remove aggregate species.

### 2.2. Analytical Size Exclusion Chromatography with Multi-Angle Light Scattering Detection (SEC-MALS)

To determine average molar mass of mAb size variants, a Waters Acquity UPLC system was used to isocratically elute 5 µg of mAb at 0.1 mL/min on a Waters SEC 200 BEH column (4.6 mm ID × 300 mm), using a mobile phase consisting of 0.20 M potassium phosphate, 0.25 M potassium chloride, pH 6.0. The effluent was directed to Wyatt uDAWN and uTrex detectors, and a data analysis was performed on ASTRA v7 software.

### 2.3. SDS-Gel Capillary Electrophoresis (CE-SDS)

Molecular weight-based separation of mAb was performed on a Beckman PA800 plus using the IgG Purity and Heterogeneity Assay Kit. Samples were diluted with SDS solution and alkylated with 40 mM iodoacetamide, followed by 5 min incubation time at 70 degrees Celsius. The detection wavelength was set at 214 nm with a separation voltage and duration of 15 KV (480 V/cm) and 40 min, respectively.

### 2.4. Differential Scanning Fluorimetry

Differential scanning fluorimetry (DSF) was used to determine the conformational stability of the hinge deleted IgG4 and of the corresponding full length mAb. Molecules were diluted to 1.0 mg/mL in 20 mM Acetate pH 5.0. Each sample was placed into three separate capillaries and tested on the NanoTemper Prometheus NT.48 nanoDSF instrument. Samples were tested at 30% excitation power and the intrinsic fluorescence was measured from 20–95 °C at a rate of 1 °C per minute.

### 2.5. Intact and Reduced Mass Spectrometry

For identification by intact MS, samples were diluted to 0.2 mg/mL in 4 M guanidine with or without reduction with 50 mM 1,4-dithiothreitol Approximately 1 µg of each sample was injected onto a Waters BEH C4 column (1.7 μm, 2.1 × 150 mm) and eluted with a linear gradient of acetonitrile containing 0.1% Trifluoroacetic acid. The column effluent was directed to a Waters G2S TOF MS instrument and raw spectra were charge deconvoluted using MassLynx 4.1 software.

### 2.6. FcγRI Binding by ELISA

IgG4 hinge deleted and full-length molecules were assessed for FcγRI binding by ELISA. Samples, reference standard and control were serially diluted and added to Neutravidin-coated High Binding Capacity microtiter plates (Thermo Scientific 15507, Waltham, MA, USA) coated with recombinant human FcγRI protein (Avi-Tag Biotinylated, Sino Biological, Catalog Number: 10256-H27H-B, Wayne, NJ, USA). Antibody binding was detected with a goat anti-human antibody conjugated with horseradish peroxidase (Jackson Immunoresearch, Catalog Number: 109-036-097, West Grove, PA, USA) and fit to a 4 PL curve. Hinge deleted sample curves were compared to that of the reference standard.

### 2.7. FcγRI Binding by Competitive AlphaScreen^®^ Assay

IgG1 hinge deleted and full-length molecules were assessed for FcγRI binding by competitive AlphaScreen^®^ assay. Samples, reference standard and control were serially diluted and added to microtiter plates (Costar 3693) containing FcγRI protein (Avi-Tag Biotinylated, Sino Biological, Catalog Number: 10256-H27H-B, Wayne, NJ, USA) conjugated AlphaScreen streptavidin donor beads (Perkin Elmer Cat # 6760002, Waltham, MA, USA). After mixing and incubation, reference standard IgG1 mAb coupled to anti human F(ab’)2goat IgG (Jackson Immunoresearch Cat # 109-006-097, West Grove, PA, USA) conjugated AlphaSceen acceptor beads (Perkin Elmer Cat # 6762001, Waltham, MA, USA) was added. After 30 min incubation, AlphaScreen^®^ signal was measured and fit to a 4 PL curve. Hinge deleted sample curves were compared to that of the reference standard.

### 2.8. FcγRIIIa Binding by ELISA

IgG1 hinge deleted and full-length molecules were assessed for FcγRIIIa V158 binding by ELISA. Samples, reference standard and control were serially diluted and added to Neutravidin-coated High Binding Capacity microtiter plates (Thermo Scientific 15507, Waltham, MA, USA) coated with recombinant human FcγRIIIa protein (Avi-Tag Biotinylated, Sino Biological, Catalog Number: 10389-H27H1b, Wayne, NJ, USA). Antibody binding was detected with a goat anti-human antibody conjugated with horseradish peroxidase (Jackson Immunoresearch, Catalog Number: 109-036-097, West Grove, PA, USA) and fit to a 4 PL curve. Hinge deleted sample curves were compared to that of the reference standard.

### 2.9. FcRn Binding by ELISA

IgG1 and IgG4 hinge deleted and full-length molecules were assessed for FcRn binding by ELISA. Samples, reference standard and control were serially diluted and added to Neutravidin-coated High Binding Capacity microtiter plates (Thermo Scientific 15507, Waltham, MA, USA) coated with recombinant human FcRn protein (Avi-Tag Biotinylated, Gilead Sciences, Foster City, CA, USA). An assay was conducted in PBS at pH 6.0. Antibody binding was detected with a goat anti-human antibody conjugated with horseradish peroxidase (Jackson Immunoresearch, Catalog Number: 109-036-097, West Grove, PA, USA) and fit to a 4 PL curve. Hinge deleted sample curves were compared to that of the reference standard.

### 2.10. Antigen Binding by Enzymatic Inhibition Assay

Relative potency of the IgG4 hinge deleted and full-length molecule samples was evaluated in a Mode of Action reflective enzymatic inhibition functional assay. All assays were carried out in a 96-well solid, black microplate (Corning, Tewksbury, MA, USA) at 24 °C. DQ^TM^ Gelatin cleavage by MMP9 was monitored by measuring the increase in fluorescence (ex: 485 nm/em: 520 nm) on an Infinite M1000 Pro Reader (Tecan, Männedorf, Switzerland) over the course of 2 h. A fixed concentration of active MMP9 (0.5 nM MMP9-MYC-6HISAPMA, 5 nM MMP9-procatAPMA or 5 nM MMP9-cat) was mixed with increasing concentrations of DQ gelatin (0–5 μM) in a final volume of 100 μL of 50 mM Tris-HCl pH 7.5, 10 mM CaCl2, 150 mM NaCl, 0.05% (v/v) Brij-35 (buffer B). The cleavage of DQ gelatin was monitored by an increase in fluorescence, and initial rates were determined. Fluorescence increase was measured continuously over 2 h. Km and Vmax were determined by fitting data to the Michaelis–Menten equation with GraphPad Prism 6. To test for residual trypsin activity, various concentrations of active MMP9-cat (0–5 nM) were mixed with a fixed concentration of DQ gelatin (100 nM) and aprotinin (0.05 mg/mL) in a final volume of 100 μL buffer B. Sample activity was expressed relative to a qualified reference standard.

### 2.11. Antigen Binding by Cell based ELISA

The relative potency of the IgG1 hinge deleted and full-length molecule samples was evaluated in a MOA-reflective functional binding assay. Samples, reference standard and control were serially diluted and added to Poly-Lysine microtiter plates coated with gp120 antigen-presenting HEK293 cells. Antibody binding was detected by fluorescence with a biotinylated donkey anti-human antibody (Jackson Immunoresearch, Catalog Number: 709-066-149, West Grove, PA, USA) followed by a europium labeled streptavidin reagent (Perkin Elmer Cat #1244-360, Waltham, MA, USA). Fluorescence signal was read on an Envision spectrophotometer (Ex/Em = 340/615 nm). The percent of relative potency was calculated from a constrained 4 PL curve fit between the reference standard and the sample.

### 2.12. Fab Arm Exchange by Förster Resonance Energy Transfer

Fab arm exchange was examined using a plate-based Förster resonance energy transfer (FRET) assay. This approach was adapted from reference 24. The hinge variant samples were chemically labeled with Dylight488, while Natalizumab (Tysabri^®^ lot 1420037,) was labeled with Dylight594. A second sample of Natalizumab was labeled with Dylight488 as a positive control. Each sample was diluted to 20 µg/mL in PBS. The antibodies labeled with Dylight488 were mixed with Natalizumab in equal volumes. These antibody mixtures were then added to a 96-well plate in either the presence (reduced) or absence (nonreduced) of reduced glutathione (GSH) at a final concentration of 1 mM. Nonreduced samples demonstrated a lack of FRET signal (Ex/Em = 490/617 nm).

### 2.13. Fab Arm Exchange by Chromatographic Separation

Fab arm exchange was also evaluated by Chromatographic separation analysis and spectrophotometric/MALS detection. This approach was adapted from reference 23. The hinge-deleted samples and Natalizumab (Tysabri^®^ lot 1420037) were diluted to 1.0 mg/mL and mixed in equal volumes in the presence and absence of 1 mM GSH. These samples were then incubated at 37 °C for 5 h. Chrom-MALS was run with a 10 µL sample injection onto a Sepax Unix SEC-300 column using a 0.2 M Potassium Phosphate, 0.25 M Potassium Chloride pH 6.0 mobile phase at a flow rate of 0.10 mL/min. Positive and negative controls were also included in the analysis. The positive control was represented by recombinant human IgG4 kappa (HCA194, BioRad), while the negative control used was recombinant human IgG4 kappa mutant (HCA247, BioRad).

## 3. Results

### 3.1. Construction and Purification of IgG1 and IgG4 Hinge Deleted Test Molecules

Two hinge deleted test molecules were built: an IgG1 with a lambda light chain and an IgG4 with a kappa light chain. The hinge sequences removed from each of the two heavy chain subtypes are shown in Figure 1. The hinge deleted molecules were expressed transiently in Chinese hamster ovary (CHO) and showed expression levels similar to that of their full-length control counterpart. All molecules were purified with a standard procedure involving a Protein A affinity step followed by gel filtration chromatography to remove aggregate species. The physicochemical and functional properties of the IgG1 and IgG4 hinge deleted mAbs and their controls were then explored.

### 3.2. Hinge Deleted Molecules Are Assembled as Monomers

**Figure 2:**
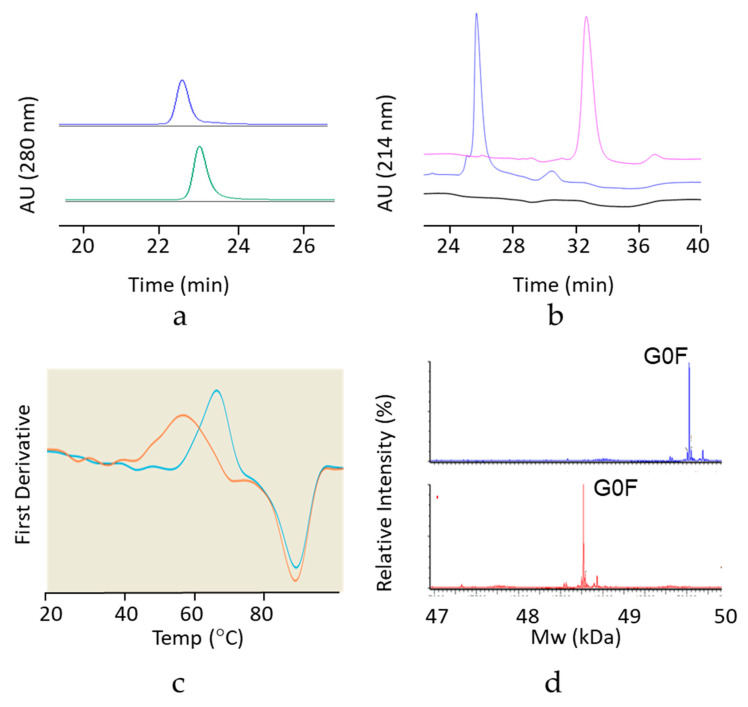
Biochemical and biophysical characterization of the hinge deleted IgG4 and its corresponding full-length control mAb by (**a**) SEC-UPLC coupled to UV (A280) and Multi Angle Laser Light Scattering (MALS). The UV elution profiles of the hinge deleted IgG4 molecule (bottom trace) and the corresponding full-length control (top trace) are shown. MALS data show measured molecular masses of 132 (hinge-deleted) and 149 kDa (full-length mAb). (**b**) Nonreduced CE-SDS coupled to UV (A280). Electropherograms of the buffer control (bottom trace), hinge deleted IgG4 molecule (middle trace) and the corresponding full-length control (top trace) are shown. (**c**) Differential Scanning Fluorimetry. An overlay of the melting profiles of the hinge deleted IgG4 molecule (orange) and the corresponding full-length control (teal) are shown. Melting temperatures/onset were 54.6/47.9 °C (hinge-deleted) and 62.7/56.6 °C (full-length mAb). (**d**) Intact reduced LC–MS. Total Ion Counts of the hinge deleted IgG4 molecule heavy chain (bottom trace) and the corresponding heavy chain of the full-length control (top trace) are shown. The peak corresponding to the dominant Fc N-Glycan G0F species is shown.

**Figure 3:**
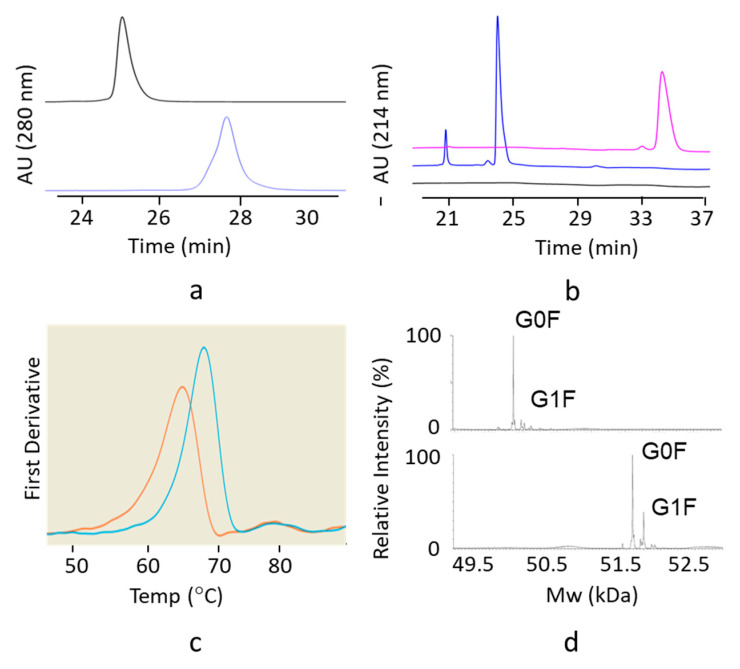
Biochemical and biophysical characterization of the hinge deleted IgG1 and its corresponding full-length control mAb by (**a**) SEC-UPLC coupled to UV (A280) and MALS. The UV elution profiles of the hinge deleted IgG1 molecule (bottom trace) and the corresponding full-length control (top trace) are shown. Molecular masses of 140 and 150 kDa were measured by MALS for the hinge deleted molecule and its full-length counterpart. (**b**) Nonreduced CE-SDS coupled to UV (A280). Electropherograms of the buffer control (bottom trace), hinge deleted IgG1 molecule (middle trace) and the corresponding full-length control (top trace) are shown. (**c**) Differential Scanning Fluorimetry. An overlay of the melting profiles of the hinge deleted IgG1 molecule (orange) and the corresponding full-length control (teal) are shown. Melting temperatures/onset were 67.8/56.5 °C for the hinge deleted molecule and 71.1/63.2 °C for the full-length control. (**d**) Intact reduced LC–MS. Total Ion Counts of the hinge deleted IgG1 molecule heavy chain (top trace) and the corresponding heavy chain of the full-length control (bottom trace) are shown. The peak corresponding to the dominant Fc N-Glycan G0F and G1F species are shown.

**Figure 4:**
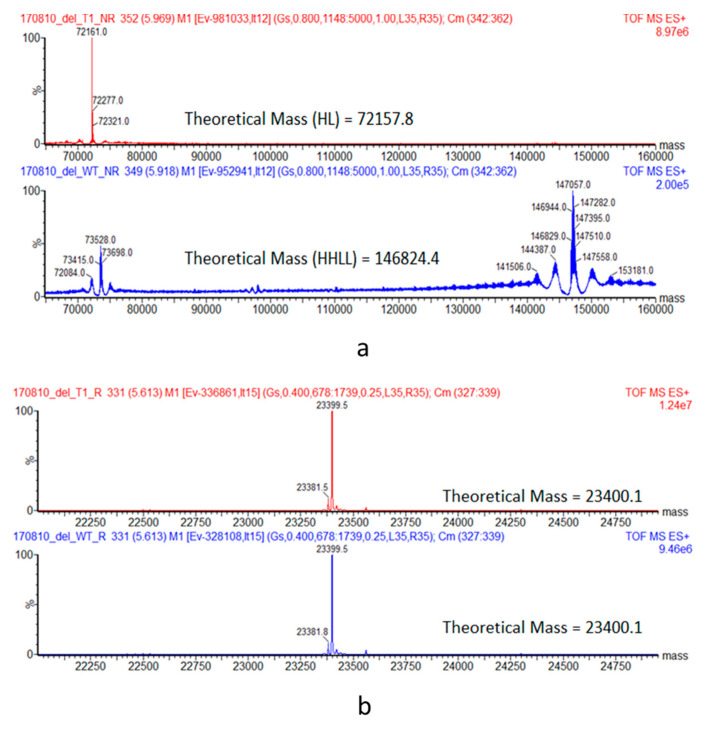
Analysis of the hinge deleted IgG4 and its corresponding full-length control mAb by LC–MS. (**a**) Deconvoluted spectrum of a nonreduced analysis. Under denaturating conditions, the hinge deleted IgG4 molecule has an apparent mass of 72,161.0 Da consistent with a Heavy-Light (HL) molecule with G0F N-Glycans. The full length mAb control has the expected mass of 146,829.0 Da consistent with the H2L2 structure (with G0F N-Glycans). Under reducing conditions, the heavy chain (Figure 3d) and Light chains (**b**) can be separated. The hinge deleted IgG4 and the full-length control molecule have the same light chain (23,399.5 Da).

The purified hinge deleted IgG1 and IgG4 mAbs were tested by size exclusion chromatography (SEC) with online UV and Multi Angle Laser Light Scattering (MALS) detectors. Under native conditions, results show the hinge truncated IgG4 molecule to have a retention time very similar to that of its full-length respective control sample (Figure 2a). The shift in retention time was more pronounced for the hinge truncated IgG1 molecule, reflecting a smaller hydrodynamic radius (4.2 vs. 5.1 nm) or possibly some interaction with the column (Figure 3a). Hinge-deleted IgG1 and IgG4 had molecular weights determined by the MALS of 140 kDa (IgG1) and 132 kDa (IgG4), consistent with a monomeric mAb quaternary structure. The two hinge deleted test molecules and their respective controls were further analyzed by nonreduced capillary electrophoresis (CE-SDS), a denaturing analytical method dissociating noncovalent protein–protein interactions (Figure 2b and Figure 3b). The CE-SDS electropherograms of the hinge deleted samples showed the presence of the expected major peak, with a migration time consistent with that of an antibody half molecule (i.e., one covalently bound heavy and light chain); the control samples displaying a slower electrophoretic mobility typical of disulfide covalently bound inter and intraheavy and light chains (Figure 2b and Figure 3b). The assignment of the molecular masses and structures to the major peaks detected in the CE-SDS electropherograms of the hinge deleted and control samples was confirmed by reversed phase and intact Mass Spectrometry (Figure 4).

### 3.3. Thermodynamic Stability of the Hinge Deleted Molecules

The impact of the hinge deletion on the thermodynamic stability of the test molecules was explored using differential scanning fluorimetry (DSF). This analytical method monitors the temperature of unfolding of the individual domains based on intrinsic fluorescence. DSF thermograms showed a melting temperature decrease of 8 °C upon deletion of the hinge of the IgG4 mAb and about 5 °C in the context of an IgG1 molecule (Figure 2c and Figure 3c). However, the melting temperature of the hinge deleted molecule was still well within the realm of thermodynamically stable proteins as the lowest temperature transition was greater than 50 °C for the IgG4 molecule and greater than 65 °C for the IgG1 (Figure 2c and Figure 3c).

### 3.4. Deletion of the Hinge Does Not Affect the Fc N-glycan Distribution

Because of the impact of the hinge deletion on the Fc thermodynamic stability, we inquired whether deletion of the hinge domain would affect the Fc N-glycan profile. Mass spectrometry results showed that the N-Glycan profile of the hinge deleted molecule was nearly identical to that of the full-length reference IgG1 and IgG4 mAbs (Figure 2d and Figure 3d). As for most mAbs expressed in CHO, the primary species attached to the hinge deleted antibody was G0F (Figure 2d and Figure 3d).

### 3.5. Hinge Deletion Obliterates Binding to Activating Fcγ Receptors I and IIIA

**Figure 5:**
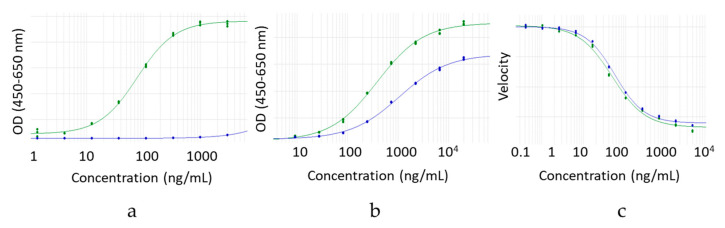
Binding of hinge deleted IgG4 (blue dilution curves) and its corresponding full-length control mAb (green dilution curves) to (**a**) FcγRI by ELISA, (**b**) FcRn by ELISA. (**c**) Enzymatic inhibition of the MMP9 target antigen.

**Figure 6:**
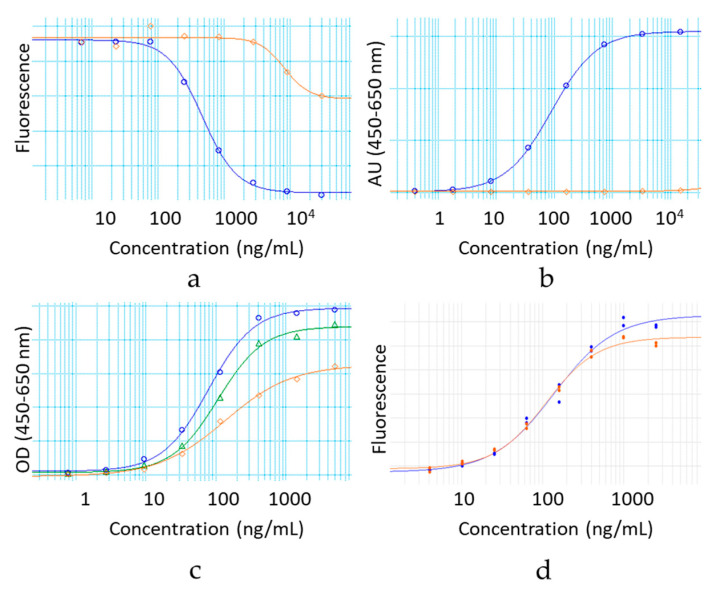
Binding of hinge deleted IgG1 (orange dilution curves) and its corresponding full-length control mAb (blue dilution curves) to (**a**) FcγRI by competitive AlphaScreen^®^ assay, (**b**) FcγRIIIa V158 by binding ELISA, (**c**) FcRn by binding ELISA. Incorporation of point mutations in the Fc of the hinge deleted IgG1 (green dilution series) almost rescues binding to the level of the full-length control. (**d**) Engagement of the gp120 target antigen by a HEK290 cell-based ELISA reflective of the mAb mode of action.

The impact of the hinge deletion on the ability of the mAbs to engage effector receptors in vitro was explored next. Monomeric IgG1 and IgG4 antibody isotypes both bind to the activating FcγRI with high affinity [19]. The binding to FcγRI of the hinge deleted IgG1 and IgG4 relative to their full-length monomeric counterpart was determined using either an ELISA (IgG4) or AlphaScreen^®^ (IgG1) assay (Figure 5a and Figure 6a). Binding to FcγRI was completely obliterated for both hinge deleted molecules (Figure 5a and Figure 6a).

FcγRIIIa binds to monomeric IgG1 isotype antibodies in the sub micromolar range and with a 10-fold relative lower affinity to IgG4 antibodies in vitro [19]. We therefore tested the impact of the deletion on the ability of the hinge deleted IgG1 test molecule to engage FcγRIIIa in a direct binding ELISA (Figure 6b). The deletion of the IgG1 hinge abolished FcγRIIIa binding, in a manner similar to what was observed for FcγRI engagement. All sets of results confirmed that for both IgG1 and IgG4, the hinge is critical for the engagement of the FcγRs, and that hinge deleted mAbs have a deactivated effector Fc domain. The loss of effector function may be explained by two potentially conjointly contributing factors. The steric hindrance of the Fab may prevent access to the FcγR binding sites on the CH2 domain. Alternatively, in a manner similar to the loss of FcγR binding caused by the structural perturbation induced by the removal of the Fc N-glycans, the destabilization of the mAb noted by DSF may reflect a structural conformation of the binding site no longer conducive to the FcγRs productive engagement.

### 3.6. Hinge Deletion Reduces Binding to FcRn

The impact of the hinge deletion on the overall Fc domain was explored by measuring the ability of the test molecules to bind FcRn. The affinity to this receptor was measured by direct binding ELISA. For both IgG1 and IgG4 molecules, the deletion of the hinge resulted in a loss of binding to FcRn (Figure 5b and Figure 6c). The lower affinity for FcRn is the most likely cause for the faster clearance of hinge deleted mAbs observed in vivo, relative to the intact molecule [18,20]. In a manner consistent with published literature, the incorporation of the M428L and N434S point mutations in the Fc of the IgG1 hinge deleted test molecules partially rescued the affinity loss (Figure 6c) [21].

### 3.7. A Point Mutation in the CH3 Domain Blocks Fab Arm Exchange in Hinge Deleted IgG4 Antibodies

**Figure 7:**
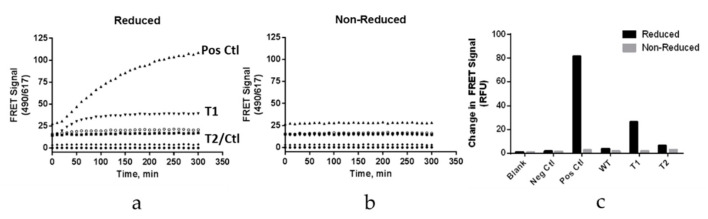
Blocking Fab arm exchange of hinge deleted mAbs by CH3 stabilization monitored by Förster resonance energy transfer analysis (FAE) which was performed under 1 mM glutathione reducing conditions (**a**) or in the absence of reducing agent as the negative control (**b**). Förster resonance energy transfer (FRET) signal (ex 490 nm/Em 515 nm) was recorded for the positive control (Natalizumab x Natalizumab), the hinge deleted IgG4 (Natalizumab x hinge deleted IgG4), the hinge deleted IgG4 with R409K point mutation in the CH3 domain (Natalizumab x R409K hinge deleted IgG4), for the negative control (S228P full length IgG4 x Natalizumab) and for the blank background control. (**c**) Bar graph summary representing the propensity of each of the test molecules to engage in FAE with Natalizumab.

**Figure 8:**
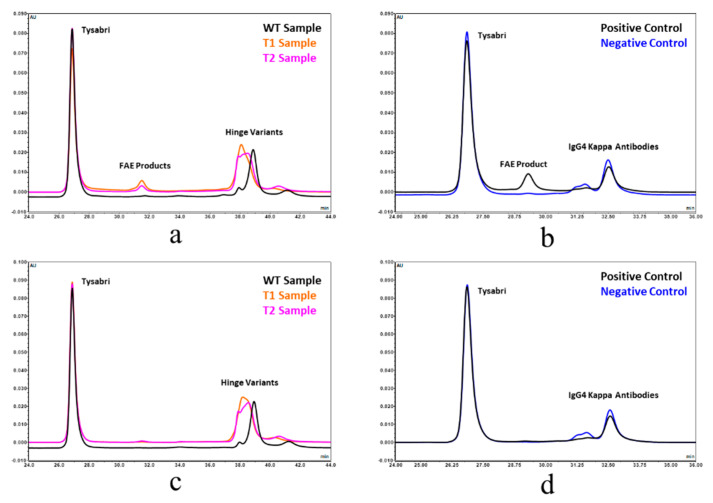
Blocking Fab arm exchange of hinge deleted mAbs by CH3 stabilization monitored by chromatographic separation. Propensity of IgG4 hinge deleted (T1), R409K CH3 stabilized IgG4 hinge deleted (T2), and S228P hinge stabilized full length IgG4 (WT) to engage in Fab arm exchange with Tysabri^®^ in the presence (**a**) or the absence (**c**) of 1 mM GSH reducing agent was analyzed by chromatographic separation. An FAE positive control reaction was conducted by monitoring the formation of the bispecific antibody by chromatographic separation between Tysabri^®^ and a nonhinge stabilized aGFP IgG4 antibody (IgG4 Kappa Antibody) in the presence (**b**) or the absence (**d**) of 1 mM GSH reducing agent.

IgG4 antibodies differ from their IgG1 counterpart in that they have the unique ability to form bispecific molecules in vivo via a process known as Fab arm exchange [1,22]. In native IgG4 molecules, the hinge disulfide bridges are in equilibrium between the inter- and intraheavy chain conformations [23]. The formation of the intraheavy chain disulfide isomer is a prerequisite for the exchange of the heavy–light chain units between two IgG4 antibodies. The molecular determinants for such a phenomenon are located in the IgG4 hinge and CH3 domain [1,8]. Modern IgG4 based therapeutic mAbs include the S228P point mutation, converting the hinge to that of an IgG1-like molecule and preventing the formation of bispecific molecules between the drug and endogenous human IgGs [1,24]. However, upon deletion of the hinge, the S228P mutation preventing FAE is removed. We therefore tested the ability of the hinge deleted IgG4 molecule to engage in FAE. This was achieved by two complementary approaches. We first relied on Förster resonance energy transfer (FRET) signal generated upon the formation of bispecific antibodies generated by the Fab arm exchange of parental antibodies labelled by two compatible fluorescent probes (Figure 7) [22,25]. Using this methodology, we tested the ability of the truncated IgG4 to exchange a half molecule (or Fab arm) with that of Natalizumab, a recombinant IgG4 with a native human IgG4 hinge. As the positive control, we conducted the exchange reaction using Natalizumab labelled with either of one of the two fluorescent probes. As the negative control, we tested the exchange of the S228P hinge stabilized full length IgG4 with Natalizumab. The results showed that the hinge deletion did enable FAE albeit to a lesser extent than when compared to the Natalizumab positive control (Figure 7). Incorporation of the stabilizing R409K mutation in the CH3 domain of the hinge deleted molecule reduced Fab arm exchange to about the same level as the negative control (Figure 7) [26]. We confirmed these observations by using a mixed mode chromatographic separation method with in line UV and MALS detectors (Figure 8) [24]. This method allowed the chromatographic separation of the two parental antibodies and their Fab arm exchanged bispecific product: the in line MALS detector yielding the molecular mass of each species eluting the column. The different test mAbs were mixed with a native hinge mAb (Natalizumab) in the presence or absence of a mild reducing agent, and each individual reaction mixture was then chromatographed (Figure 8). The results showed the formation of a ~140 kDa hinge deleted mAb-Natalizumab hybrid eluting between the two parental antibodies (Figure 8) [24]. As before, the R409K stabilization of the CH3 domain compensated for the absence of the S228P mutation in the hinge. In this assay format, positive and negative controls resulted in the expected formation of bispecific antibodies or their absence (Figure 8).

### 3.8. Hinge Deletion Does Not Alter Antigen Binding

Lastly, the impact of hinge deletion on antigen recognition and binding was explored by determining the relative potency of the hinge deleted test molecules. The IgG1 and IgG4 antibodies were tested against their respective target antigen. The hinge deleted IgG4 molecules were tested using an enzymatic inhibition functional assay, which showed the deletion of the hinge to be innocuous to the antibody Fab function (Figure 5c). Likewise, relative binding affinity of the hinge deleted IgG1 determined by cell-based ELISA showed no impact of the deletion on the ability to bind its cognate antigen (Figure 6d).

## 4. Discussions and Conclusions

The last twenty years have seen the full realization of IgG potential as therapeutic molecules to treat a wide array of ailments such as inflammation, infectious diseases and hematological and solid tumor cancers [27]. The new generation of therapeutic mAbs in clinical development incorporates molecular engineering derived from the most recent advances in the understanding of the interplay of immunoglobulins with the different receptors and interacting partners [14]. Molecular engineering is intended to tailor the function of these therapeutic mAbs to their specific mode of action and enhance their therapeutic index by increasing their efficacy or reducing undesirable safety signals.

A great deal of effort has been particularly devoted to addressing the potential safety liability associated with the Fc effector function when it is not required as part of a mAb mode of action. A number of Fc silencing strategies such as the removal of the Fc glycosylation or the introduction of point mutations at specific locations in the lower hinge of the Fc have been proposed [14]. These strategies, although clinically validated in some cases, may not offer a complete Fc passivation and may only affect the binding of some of the FcγRs or may create potentially immunogenic neo-epitopes.

The alternative Fc passivation strategy we propose in this report relies on the complete deletion of the hinge of a therapeutic mAb. Our results concur with earlier published data, establishing FcγRI, FcγRIIIA and C1q binding inactivation [16,17,18]. In addition to impacting FcγR binding, deletion of the hinge greatly reduces binding to FcRn, which results in a significant shortening of the molecule in vivo half-life [18,20]. The mechanism linking hinge deletion and impact on FcRn binding is unclear. However, the abrogation of FcγR binding and reduction in FcRn binding, which have different binding sites, suggest the alteration of the CH2 domain conformational stability or dynamics. While both hinge deletion and Fc aglycosylation result in the thermodynamic Fc destabilization of the same magnitude (~6–8 °C), the removal of Fc N-glycans does not appear to impact FcRn binding [28]. This potentially suggests a structural perturbation of a different nature in both cases.

The concomitant abrogation of the effector function and half-life reduction constitutes a particularly appealing proposition for the design of molecules with an improved safety profile and exposures intermediate between mAb fragments and full IgGs. The presence of an Fc domain prevents renal glomerular filtration and still ensures half-lives significantly longer than the smaller Fabs or single chain Fv constructs [20]. If deemed necessary, simple point mutations in the FcRn binding site allow for the modulation of the pharmacokinetic properties of the molecule to nearly match that of a standard mAb.

Owing to their particular functional and pharmaceutical properties, hingeless antibodies could find their place in the therapeutic arsenal to address a number of niche applications. The effectorless properties of a hinge deleted IgG4 mAb have recently been presented as a potent blocking agent for the treatment of neuromuscular autoimmune disease myasthenia gravis in rhesus monkeys [18]. Beyond the more traditional blocking mode of action, the effectorless and shorter pharmacokinetics properties of recombinant mAbs could also be appealing for an array of different applications including the activation of cell surface receptors by Fc independent direct agonists when a pharmacodynamic effect does not need to be sustained via prolonged exposure or as an in vivo biologically inert diagnostic imaging agents with a clearance rate intermediate between those of a Fab and a full length monomeric mAb [29]. Hinge truncation also offers the opportunity of generating protease resistant mAb based therapeutics for the inactivation and clearance of toxins of bacterial origin during bacterial infections [30].
